# Developing a Tooth *in situ* Organ Culture Model for Dental and Periodontal Regeneration Research

**DOI:** 10.3389/fbioe.2020.581413

**Published:** 2021-01-18

**Authors:** Reem El-Gendy, Sarah Junaid, Stephen K. L. Lam, Karen M. Elson, Joanne L. Tipper, Richard M. Hall, Eileen Ingham, Jennifer Kirkham

**Affiliations:** ^1^Division of Oral Biology, School of Dentistry, University of Leeds, Leeds, United Kingdom; ^2^Department of Oral Pathology, Faculty of Dentistry, Suez Canal University, Ismailia, Egypt; ^3^School of Engineering and Applied Sciences, Aston University, Birmingham, United Kingdom; ^4^School of Mechanical Engineering, University of Leeds, Leeds, United Kingdom; ^5^Tissue Engineering Group, Faculty of Biological Sciences, School of Biomedical Science, University of Leeds, Leeds, United Kingdom; ^6^Institute of Medical and Biological Engineering (IMBE), University of Leeds, Leeds, United Kingdom; ^7^School of Biomedical Engineering, University of Technology, Sydney, NSW, Australia

**Keywords:** organ culture, tooth *in situ*, dental regeneration, periodontal, bioreactor, simulation model, periodontium

## Abstract

In this study we have realized the need for an organ culture tooth *in situ* model to simulate the tooth structure especially the tooth attachment apparatus. The importance of such a model is to open avenues for investigating regeneration of the complex tooth and tooth attachment tissues and to reduce the need for experimental animals in investigating dental materials and treatments in the future. The aim of this study was to develop a porcine tooth *in situ* organ culture model and a novel bioreactor suitable for future studies of periodontal regeneration, including application of appropriate physiological loading. The Objectives of this study was to establish tissue viability, maintenance of tissue structure, and model sterility after 1 and 4 days of culture. To model diffusion characteristics within the organ culture system and design and develop a bioreactor that allows tooth loading and simulation of the chewing cycle.

**Methods:** Twenty-one porcine first molars were dissected aseptically *in situ* within their bony sockets. Twelve were used to optimize sterility and determine tissue viability. The remainder were used in a 4-day organ culture study in basal medium. Sterility was determined for medium samples and swabs taken from all tissue components, using standard aerobic and anaerobic microbiological cultures. Tissue viability was determined at days 1 and 4 using an XTT assay and Glucose consumption assays. Maintenance of structure was confirmed using histology and histomorphometric analysis. Diffusion characteristics were investigated using micro-CT combined with finite element modeling. A suitable bioreactor was designed to permit longer term culture with application of mechanical loading to the tooth *in situ*.

**Result:** XTT and Glucose consumption assays confirmed viability throughout the culture period for all tissues investigated. Histological and histomorphometric analysis confirmed maintenance of tissue structure. Clear microbiological cultures indicated maintenance of sterility within the organ culture system. The novel bioreactor showed no evidence of medium contamination after 4 days of culture. Finite element modeling indicated nutrient availability to the periodontium.

**Conclusion:** A whole tooth *in situ* organ culture system was successfully maintained over 4 days *in vitro*.

## Introduction

Periodontal disease (Chenery, 2011) is the sixth most common diseases across the world (Murray et al., 2012) Acute to severe periodontitis affects some 64.7 million people in the US alone, 20% of the population and 47% of the population aged over 30 (Eke et al., 2012). In England, there is a 45 per cent prevalence of mild to advanced PD and 9 per cent prevalence of advanced PD (Chenery, 2011), which commonly leads to tooth loss. PD may be described as a multi-factorial chronic inflammatory disease that arises when persistent microbial accumulations (plaque) at the tooth-gingiva interface invoke a host response that can lead to destruction of the tooth attachment apparatus (the “periodontium”) (Nield-Gehrig, 2008). PD severity ranges from bleeding of the gums (gingivitis), to loss of tooth attachment due to destruction of the periodontal ligament, loss of alveolar bone and ultimately to loss of the tooth. There are increasing numbers of reports of links between PD and systemic disease, including illnesses as diverse as rheumatoid arthritis and Alzheimer's disease [for example (Kamer et al., 2008; Berthelot and Le Goff, 2010)]. In addition, the symptoms of PD can be aggravated by patients' systemic conditions, such as type 1 and type 2 diabetes mellitus (Grossi and Genco, 1998; Highfield, 2009; Al-Mendalawi, 2010; Hsu et al., 2019). Surgical intervention is required when PD has progressed and is associated with mineralized tissue loss including alveolar bone defects. These types of surgical procedures are carried out in attempt to stimulate repair of the periodontium. However, bone grafting and guided tissue regeneration are required in more advanced three-walled osseous defects. Membrane guided regeneration has been successful when combined with regenerative agents such as enamel matrix protein (Pihlstrom et al., 1983; Dentino et al., 2013). Many of the current multiphasic treatments for PD are successful, at least in the short term but they are limited to repair rather than regeneration. Even guided tissue regeneration (GTR) has associated complications, including exposure of the supporting membrane leading to subsequent infections in 50% of cases (Villar and Cochran, 2010). The clinical need for successful long term regeneration of the periodontium remains and future treatment strategies based on tissue engineering approaches offer some new hope (Villar and Cochran, 2010; Liu, 2019). In worst case scenario an implant surgery might be required to replace lost tooth. The success of this line of treatment would require osseointegration and successful bone regeneration around the implant, which can also be affected by patients' general health and comorbidities (Albrektsson and Albrektsson, 1987; Sarve et al., 2016; Guglielmotti et al., 2019). Successful implants in patients with co-morbidities such as osteoporosis can require bone grafts or more advanced bone regeneration strategies (Erdogan et al., 2007; Vinci et al., 2019).

Current pre-clinical testing models for bone, dental and periodontal regenerative strategies have recognized limitations. *In vitro* models, usually cell monolayers, lack the organizational complexity and physiological loading experienced by the periodontal tissues *in vivo* (Pavlin and Gluhak-Heinrich, 2001; Chukkapalli and Lele, 2018). In the case of animal models, tooth morphology and prevalent loading forces, together with the anatomy of the periodontal attachment are frequently unrepresentative of the human periodontium (Ismaiel et al., 1989; Dannan, 2007; Struillou et al., 2010; Oz and Puleo, 2011). Furthermore, animal models may be associated with ethical controversies and are relatively expensive to deliver. Current *ex vivo* models include tooth slice and mandibular slice models (Smith et al., 2010; Sloan et al., 2013). In spite of these models being extremely successful in simulating dentine and bone healing and regeneration in health and disease (Smith et al., 2010; El-Bialy et al., 2011; Wan Hassan et al., 2012; Sloan et al., 2013), they remain limited to what is essentially a section of the periodontal ligament.

The ultimate goal of this research was to develop a porcine tooth *in situ* (i.e., within its bony socket) organ culture model that could ultimately be used within a bioreactor designed to provide appropriate mechanical stimulation to the tissues by simulation of chewing cycles and orgnathic forces. The aim of the current study was to take the first step toward achieving this model by developing and validating an organ culture system capable of maintaining viability and sterility of an intact porcine tooth *in situ* for up to 4 days, as well as designing a bioreactor for future applications.

## Materials and Methods

### Tissue Procurement

**Figure 1:**
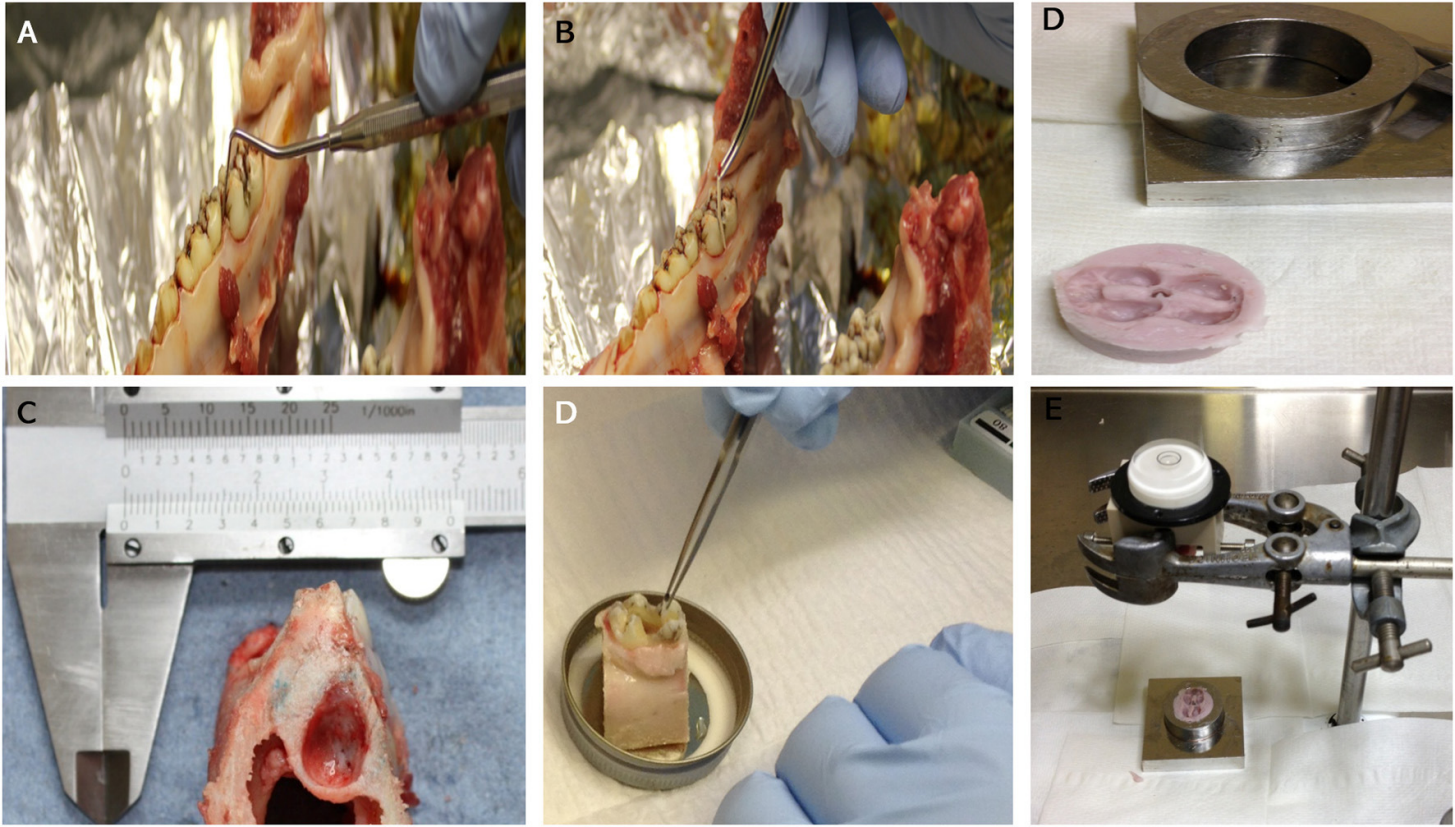
An image showing: Measuring of periodontal pocket depth around the first molar prior to dissection **(A)**: taking swabs from the gingival crevice before **(B)** and after cleaning **(D)**. General appearance of the molar tooth *in situ* within the socket after dissection (lateral view) **(C)**. Images **(E,F)**: dental putty impression of the tooth crown that is used as a cap for the crown part of the tooth *in situ* model to receive the loading within the bioreactor.

Mandibles containing teeth *in situ* were isolated from the heads of Yorkshire White pigs, 22- 26 weeks of age, within <2 h of slaughter at the local abattoir. A dissection process to obtain a tooth *in situ* within the mandible was carried out under aseptic conditions in a class II laminar flow hood; any tissues that were not included in the dissection were covered in sterile drapes. The first molars on each side of the mandible were isolated within their bony sockets using a highspeed electric saw irrigated with chilled PBS during the cutting procedure. Standardization of size and cut surfaces of each tooth *in situ* sample was achieved using a template rig especially designed in-house for this purpose (Figure 1A). Sample size and weight and periodontal pocket depth were recorded for each tooth *in situ* sample and any samples with a periodontal pocket depth > 3 mm were excluded (Figure 1B). Samples were cleaned using an electric toothbrush and toothpaste, washed thoroughly using a water pick and scaled with a dental scaler to remove any food debris or calculus deposits. To avoid pulp necrosis during the organ culture period and to aid perfusion, pulp tissue was extracted completely from the pulp chamber and root canals via surgical pulpectomy using sterile surgical burs (rounded and fissured stainless steel surgical burs size one) and endodontic files (Kerr stainless steel K file lengths 21–25 mm sizes 55–80).

### Organ Culture of Tooth *in situ* Samples

The average overall dimensions of the harvested tooth *in situ* samples (including the surrounding gingiva and bone) was 20 mm (L) × 30 mm (W) × 35 mm (H) ± 1.5 mm (Figure 1A). The average weight of the samples was 13.04 ± 0.95 g. After harvesting, the tooth *in situ* samples were subjected to further washing, first in 0.2% chlorhexidine digluconate, alcohol free mouth wash solution (Corsodyl®) diluted 1:1 in PBS for 10 min and then in PBS containing antibiotics [100 U/mL penicillin/streptomycin (Lonza DE17-603E) + 2.5 μg/mL amphotericin B (Sigma A2942)+ 50 μg/mL gentamycin (Sigma G1272)] for 30 min. The samples were then pre-cultured in medium [high glucose DMEM supplemented with 20% FBS (Lonza)], 100 U/mL penicillin/streptomycin [Lonza DE17-603E), 2.5 μg/mL amphotericin B (Sigma A2942) and 50 μg/mL gentamycin (Sigma G1272)] overnight at 37°C and 5% CO_2_. The medium was changed the following day and then every other day for 4 days under the same culture conditions.

### Optimization of Antibiotics to Minimize Infections in the Culture

In order to identify best candidate antibiotic mixes and optimize their concentrations in our cultures, anti-microbial susceptibility tests (Berendsen et al., 2009) were carried out using the disc diffusion method (Reller et al., 2009) on lawns from bacterial isolates cultured on Muller Hinton agar (Sigma−70192) plates under aerobic (37°C and 5% CO_2_) and anaerobic (37°C in an [Whitely A45 Anaerobic work station (Don Whitely Scientific, UK)], at 10% CO_2_, 80% N_2_, 10% H_2_, culture conditions. The minimum inhibitory concentrations (Murray et al., 2012) were also determined using the turbidity determination method (Reller et al., 2009). Briefly, serial dilutions of the antibiotics are added to equal volumes of broth (5 ml), then 10 μl of the inoculum are added to broth/antibiotic mix. In order to identify the lowest antibiotic concentrations required to prevent visible microbial growth in an overnight incubation MIC was determined at ≥ 80% reduction in growth compared to controls (Reller et al., 2009). Isolates were obtained by swabbing different regions of the tooth *in situ* dissected samples using sterile endodontic paper points (Dentsply, UK), the MIC was determined for each of the following antibiotics using the turbidity determination as explained previously (Erythromycin, Fusidic acid, Gentamycin, Penicillin G, Tetracycline, Vancomycin) for the bacterial isolates obtained.

### Confirmation of Infection Control in the Tooth *in situ* Organ Cultures

After determination of optimum antibiotic mix, organ cultures were then carried out overnight and for up to 4 days using the selected mixture of antibiotics [100 U/mL penicillin/streptomycin (Lonza DE17-603E), 2.5 μg/mL amphotericin B (Sigma A2942) and 50 μg/mL gentamycin (Sigma G1272)] and the same media described in section Optimization of Antibiotics to Minimize Infections in the Culture. Confirmation of infection control in cultures was carried out using standard microbiological cultures for aerobic and anaerobic bacteria and yeasts on agar plates, using tissue swabs taken from the different regions of the tooth *in situ* samples as described above, both before and after the cleaning procedures (Figure 1).

### Determination of Tissue Viability

Tooth *in situ* samples were dissected and cleaned as described previously. The viability of periodontal tissues from 2 samples harvested from a single animal (one from each side of the mandible) were used for each experiment. Experiments were repeated 3 times using samples obtained from 3 different animals.

#### Determination of Tissue Viability Using an XTT Assay

Porcine tooth *in situ* samples were dissected to separate out individual tissue components comprising of gingivae, PDL, alveolar bone and cortical bone and the viability of each weighed tissue was determined using the XTT assay (Sigma Tox2-1 kit) according to manufacturer's instructions with slight modification to suit the nature of tissues investigated (Elson et al., 2015). Briefly, pre-weighted tissues (gingivae, PDL, alveolar bone and cortical bone) were chopped into ~2 mm^3^ pieces and incubated in the XTT solution (1 mL) for 4 hours at 37°C in 5% (v/v) CO2 in air and the XTT solution removed and retained. Tetrazolium product was then extracted from the tissues with dimethyl sulfoxide (DMSO; 0.5 mL; VWR Chemicals, Lutterworth, UK; product code 23500.297) for 1 h. XTT and DMSO solutions were then pooled before reading the absorbance of triplicate samples at 450 and 690 nm in a 96 well plate (Nunc A/S [Thermo Fisher Scientific], Roskilde, Denmark; product code 269620) using a microplate spectrophotometer (Thermo Scientific, Fisher Scientific, Loughborough, UK; model Multiskan Spectrum) and SkanIt™ RE for MSS 2.1 software (Thermo Software, Thermo Scientific). The absorbance at 690 nm were subtracted from those at 450 nm and calculated per gram of tissue. The readings were compared after 1 and 4 days of organ culture.

#### Glucose Consumption by Porcine Tooth *in situ* Samples During Organ Culture

Glucose consumption levels were measured in samples of conditioned medium obtained from the tooth *in situ* organ cultures using a GlucCell® Monitoring System (Cesco Bioengineering, Taiwan) as per the manufacturer's instructions.

Briefly, fresh and conditioned culture medium samples (30 μL) were pipetted onto a clean hydrophobic surface in duplicate and measured using glucose test strips (Cesco Bioproducts; product code DGA050) according to the manufacturer's instructions. Average readings were used to calculate total glucose concentration in each sample. The concentrations of glucose in the conditioned medium were subtracted from the concentration of glucose in the fresh medium to determine the amount of glucose used (mg). This was then divided by the tissue weight (g) and again by the number of days in culture to give glucose (mg) utilized per gram of tissue per day in culture (Elson et al., 2015). This permitted comparisons to be made between glucose consumption after 24 h (1 day) pre-incubation in medium containing the antibiotic mix to glucose consumption in the same tooth *in situ* samples (*n* = 3) after 4 days of organ culture.

### Histology and Image Analysis

Tooth *in situ* samples (Figure 1B), either freshly prepared (immediately following dissection from the mandibles; *n* = 3) or following 4 days of organ culture (*n* = 3), were fixed by immersion in 10% neutral buffered formalin (NBF) before being cut longitudinally (corono-apical direction from the proximal aspect), into 1.5 mm thick slices, using Accutome 5 section cutter (Struers, Ballerup, Denmark). Slices were demineralised in 14% EDTA (pH 7.4), for 3 weeks, with shaking, at room temperature. EDTA solution was changed every 2–3 days. After complete demineralisation was confirmed by X-ray images, samples were paraffin embedded and sectioned longitudinally to produce 5 μm sections prior to being stained with haematoxylin and eosin and viewed using a light Nikon microscope. Micrographs were image analyzed using Nikon Elements System (Murray et al., 2012) software, version 3 (Tokyo, Japan). Three random fields in each of 3 sections for 3 different tooth *in situ* samples and corresponding freshly prepared controls samples isolated from the same animals, were used. Each field represented the central, mesial and distal surfaces of the samples. The histological appearance of the gingivae, PDL and alveolar bone was compared for all samples (a total of 36 fields/tooth *in situ* or fresh control sample). In addition, PDL thicknesses were measured at mid root, along with total numbers of cells and blood vessels in PDL and alveolar bone. The resulting data was compared for tissues obtained from the organ cultures with those obtained from freshly prepared controls.

### Modeling Diffusion Based on Tissue Porosity

**Figure 2:**
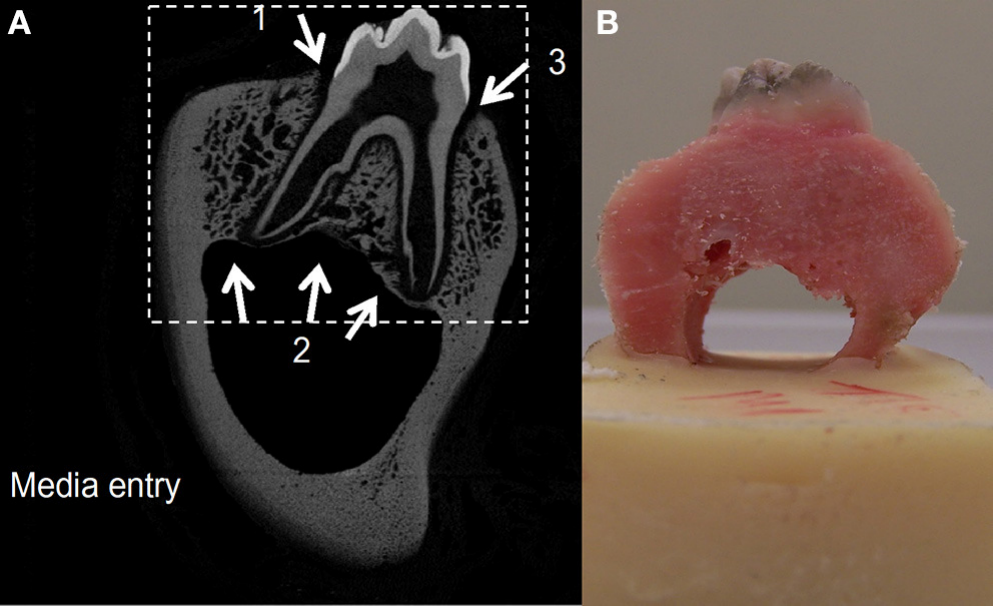
A two-dimensional slice from the CT scan of a porcine premolar tooth. Greyscale is proportional to the density of the bone, hence image is used as a porosity map where black depicts extracellular space and gray shading depicts varied solid bone densities **(A)**. A porcine premolar tooth before ink diffusion test **(B)**.

In order to obtain information on the possible diffusion pathways for nutrients, oxygen, and metabolic waste within the organ culture system, image analysis was carried out to produce a tissue diffusion map. A porcine molar tooth with the surrounding periodontal ligament and intact bone was dissected from the mandible as described previously and scanned using micro-computed tomography (microCT; MicroCT100, Scanco Medical, Brüttisellen, Switzerland) at 49.2 μm resolution (test parameters: 70 KVp, 114 μA, 300 ms). A microCT image slice was taken from the coronal plane for image processing (Figure 2). Image processing was carried out using photo editing software (Coral PaintShop Pro Photo XI, Ottawa, Canada) and Matlab (Mathworks, Natick, MA, USA) in order to create a two-dimensional map combining bone porosity from the microCT image and contour lines representing tissue depth. The tissue depth contour lines reflected the distance from the gingival epithelium or mandibular canal. The porosity map from the microCT image was combined with the tissue depth contour map to create a combined tissue diffusion map that would predict the diffusion of medium in areas known to be difficult to perfuse due to bone thickness and higher bone density.

The cortical bone (cortical shell) of the mandible surrounding the alveolar bone was assumed to be impermeable externally therefore no diffusion was modeled from the external cortical shell. Three inlet diffusion points into the tissue were identified as two points at the junctional epithelium and one at the inferior alveolar canal. Contour mapping was carried out from these three inlet points with each contour representing 1 mm. The percentage porosity for each greyscale pixel was calculated using Matlab, with cortical bone and air representing the two ends of the scale (zero porosity and 100% porosity, respectively).

The porosity and contour maps were combined to produce a weighted contour map for each inlet by using the formula:

$$\begin{array}{l} •\sum \frac{Contourband\;\#\;\times \;Pixel\;size\;\left(mm\right)}{Porosity\left(\%\right)} \end{array}$$

The formula demonstrates that in any region, the lower the porosity (i.e., the denser the bone), then the higher the number of contour bands. The three inlet maps were combined to produce a single porosity weighted map.

The tissue diffusion contour map was tested experimentally for validation using porcine tissue and an Indian ink test in PBS solution. Four porcine first molar teeth were dissected from the mandibles and cleaned. The lower portion of the mandibular canal was embedded in polymethylmethacrylate (PMMA) bone cement (Figure 2) and placed in an Indian ink solution (one part ink to three parts PBS) and left to diffuse for 18–21 h. The dye was washed out with PBS and the bone was cut in the coronal plane using a diamond tipped high precision bone saw (Exakt bonesaw, OK, USA).

### Bioreactor Design

**Figure 3:**
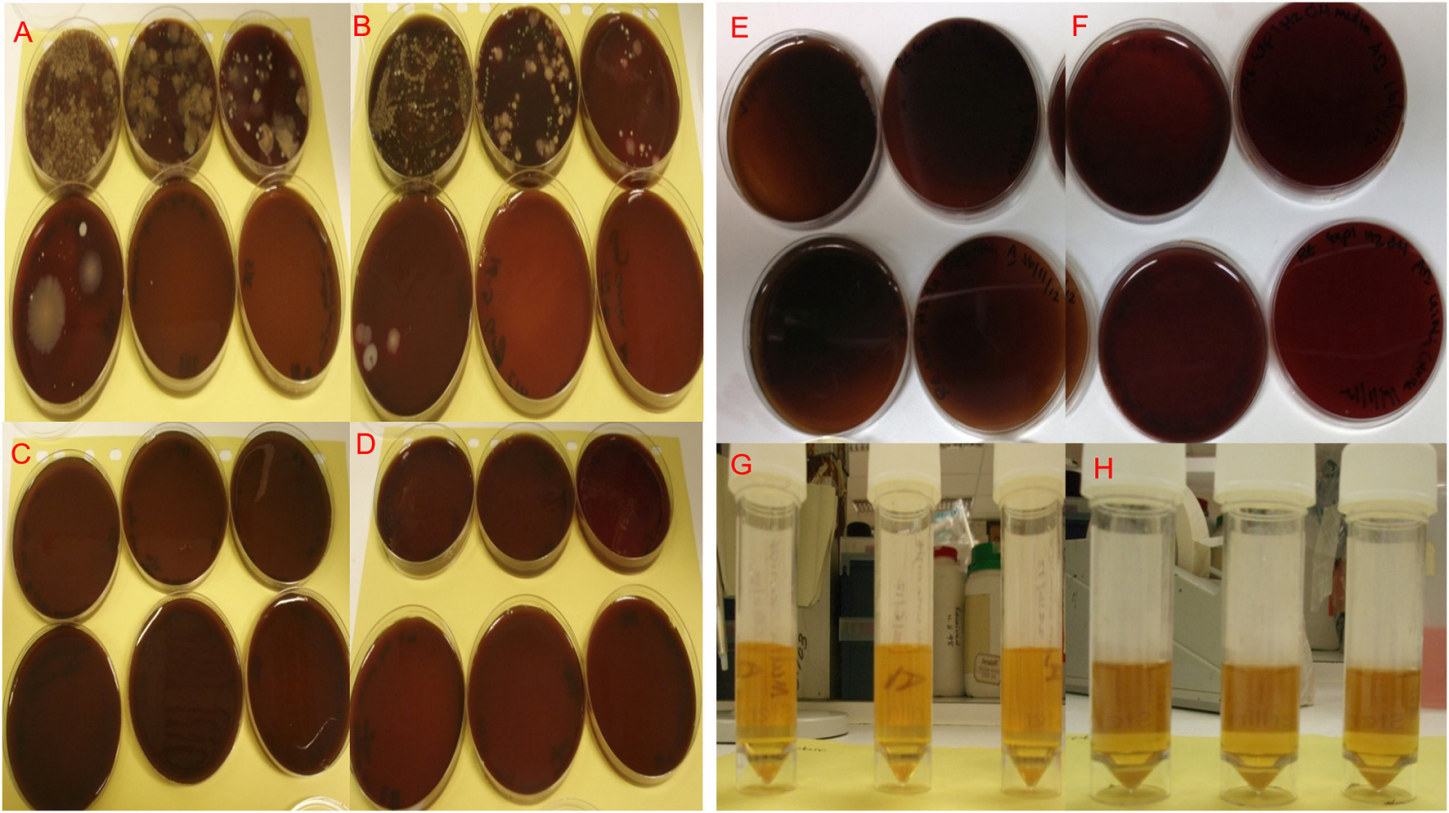
Microbiological cultures of bacterial isolates from paper point swabs taken from organ culture teeth *in situ* in freshly dissected samples before cleaning and washing, the bacterial isolates were serially diluted (10^−1^–10^−6^) and cultured under aerobic **(A)** and anaerobic **(B)** conditions. Cultures of bacterial isolates from paper point swabs taken from the organ culture after cleaning and washing in antibiotics overnight were serially diluted (10^−1^–10^−6^) and cultured under aerobic **(C)** and anaerobic **(D)** culture conditions, showed complete absence of infection in plates cultured after cleaning and overnight washing in antibiotics. Aerobic and anaerobic cultures for paper point swabs taken form teeth *in situ* **(E)** and aerobic and anaerobic growth broth for media used to culture the tooth *in situ* **(F)** confirming the absence of contamination and bacterial infections after 4 day in culture. Isolates were cultured under Aerobic conditions are shown in **(A,C,E,G)** and under anaerobic conditions in **(B,D,F,H)**.

The bioreactor unit was machined from Polyetheretherketone (PEEK) and sterilized by autoclave. The design consisted of an outer container and an internal detachable porous specimen holder where the *in situ* samples were seated after the tissue cleaning process and secured using grub screws. The specimen's holder was designed to mimic the inferior alveolar canal where nutrient exchange occurs physiologically (Figures 3A,B). The bioreactor unit contained an inlet and outlet (Figure 3C), at the base for media change and media sampling, which was carried out every 48 h until the test was terminated. To transmit mechanical loading through chewing forces, a mold of the tooth was made after pulpectomy using a silicon dental impression material (putty) (Zhermack, Badia Polesine, IT) (Figures 1D,E), which was then sterilized via autoclave and placed as a loading cap on the specimen in the bioreactor. The silicon mold was in the shape of the crown of the tooth, thus enabling a distributed load, which is believed to be more conducive to chewing than a point load. An opening through the center of the loading cap allowed for media to enter the root canal of the tooth. The bioreactor was sealed using a permeable semi-transparent membrane (OPSITE®, Smith and Nephew, Hull, UK) (Figure 3C) to maintain a sterile environment. The loading through the loading cap was therefore possible with the OPSITE®, as a sterile barrier between the mechanical loading system and the loading cap and tooth.

The mechanical loading was applied through a flat platen attached to a uniaxial mechanical loading frame (Instron, Norwood, MA, USA) (Figure 3D). The tooth was pre-cycled between −5 to −45 N at 0.25 Hz for 10 cycles then loaded at 1 Hz from −5 to −100 N ± 2.6% sinusoidally for 1,400 cycles. To test the mechanical integrity of the silicon loading cap, the loading regime was carried out where the loading cap was placed on a tooth structure in a non-sterile test and mechanically loaded as described. One sample was successfully loaded.

A sterility test was carried out with media and no specimen, testing sterility of the bioreactor system and silicon loading cap over a 4-day period. Swabs were taken at different locations and components of the system (silicon loading cap, inlet, outlet, specimen holder, and bioreactor chamber) for aerobic and anaerobic bacterial culture. Furthermore, sterility against aerobic and anaerobic bacteria was carried out for one *in situ* sample under loading.

Verification of tissue viability in the tooth *in situ* model after 4 days of culture with the dental putty cap and within the bioreactor has been confirmed using XTT for all tissues.

### Statistical Analysis of Data

Tissue viability data (XTT and glucose consumption) and histomorphometric analyses were analyzed using Anova followed by Tuckey's *post hoc*-test in Graph Pad prism software version 6.

## Results

### Infection Control in Organ Cultures

**Figure 4:**
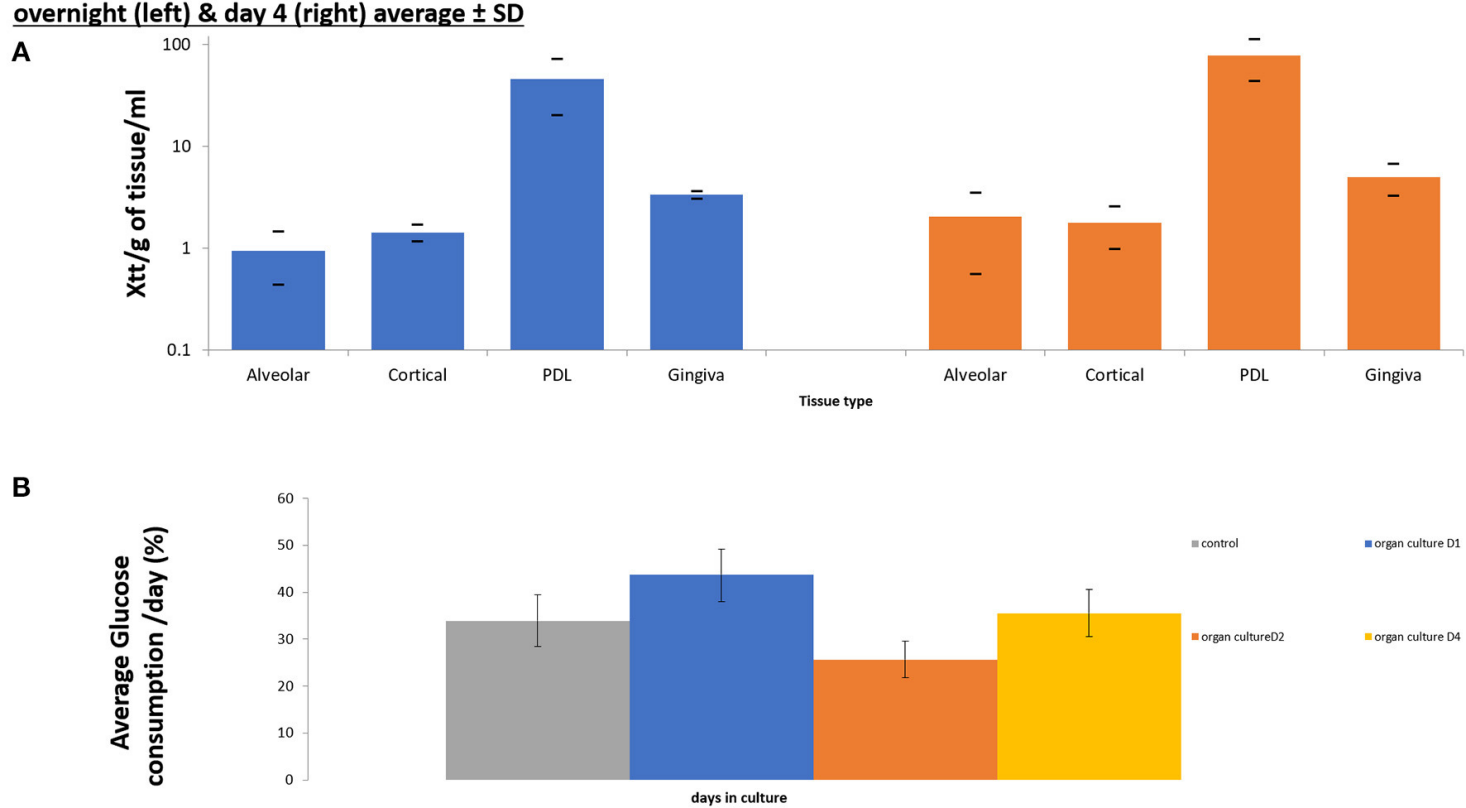
Cell viability assays confirming maintenance of cell viability in all components of tooth *in situ* organ culture (alveolar bone, compact bone, PDL, and gingiva) after 4 days of culture compared to overnight control samples using XTT viability assay (*n* = 3) **(A)**. Glucose consumption assay calculating percentage of glucose consumption/ day of culture and confirming viability of the organ culture compared to overnight (D0) controls (*n* = 3) **(B)**.

Levels of infection before cleaning (controls) and after organ culture for 1 and 4 days are shown in Figure 4. The selected mixture of antibiotics at defined concentrations identified following the MIC and AMST assays proved able to maintain high levels of infection control as evidenced by in Figure 4. The initial microbial load (isolated by swabbing the different areas of the organ culture as described in section Confirmation of Infection Control in the Tooth *in situ* Organ Cultures and Determination of Tissue Viability) before cleaning, was 38 × 10^4^ colony forming unit (CFU)/mL in isolates cultured from swabs under Aerobic conditions, whereas under Anaerobic conditions, the microbial loads were 33 × 10^4^ CFU/mL. Our optimized method of cleaning and the optimized antibiotics choice and concentration have shown to eliminate aerobic and anaerobic infections in 90% of the cases after overnight culture and up to 4 days in culture (zero CFU/mL) Figure 4. Ten percent of the samples showed a mixed aerobic and anaerobic infections during or at the end of the 4 days culture.

Yeast cultures were negative both before and after cleaning in 100% of the tooth *in situ* organ culture samples.

### Confirmation of Tissue Viability of the Organ Culture

XTT absorbance at 450 nm after subtracting the background at 690 nm were calculated /g of tissue /ml of media. The XTT measurement indicated the production of Tetrazolium by the tissues as an indication of viability and metabolism. Xtt was measured in overnight controls and in 4 days organ cultures. The XTT was measured separately for each tissue that comprised the porcine tooth *in situ* model (Gingiva, PDL, Alveolar and cortical bone).

**Figure 5:**
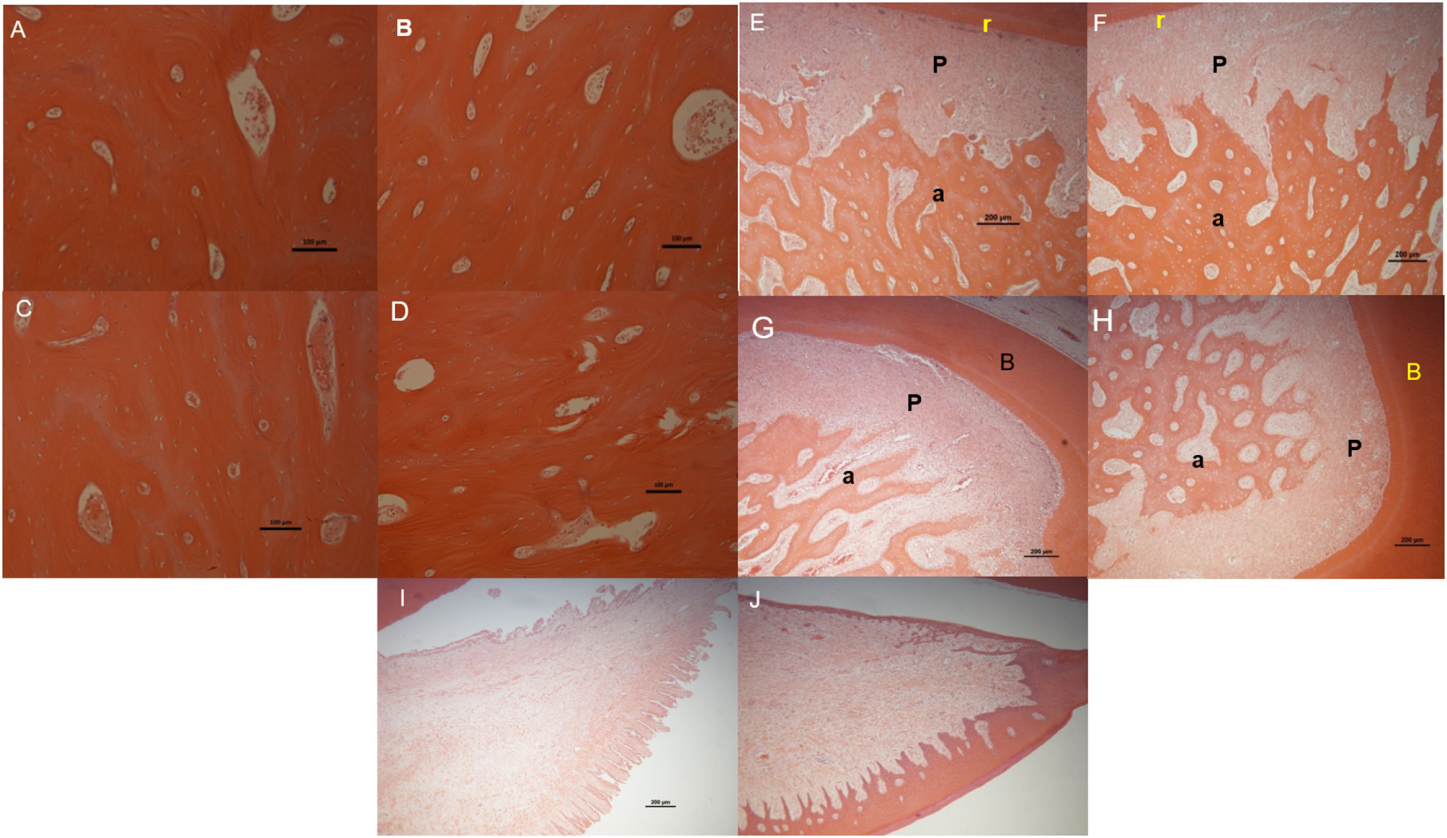
Light microscope images of H&E stained sections for the following tissues/regions: buccal bone plate in fresh control samples **(A)** and in organ culture tooth *in situ* (4 days of culture) samples **(B)**, lingual bone plates in fresh control samples **(C)** and in organ culture tooth *in situ* samples **(D)**, PDL (P) attached between tooth root (r) and alveolar bone (a) in fresh control samples **(E)** and in organ culture tooth in situ samples **(F)** and periodontal attachment at the roots bifurcation (b) area in fresh control samples **(G)** and in tooth *in situ* organ culture **(H)**. Gingival tissue in fresh controls **(I)** and in organ culture test samples **(J)**. All images have shown maintenance of structure in tooth in situ after 4 days in culture compared to fresh control samples from the same animal except for gingival tissue in which the epithelium was stripped during histological preparation.

There was no significance difference in XTT absorbance/g tissue/mL of media, between tissues obtained from tooth *in situ* samples that had been in organ culture for 4 days and controls isolated form the same animal, that were incubated in pre-culture (overnight only) (Figure 5A). However, PDL showed the highest cell viability of all tissues followed by the gingiva in controls and after 4 days of culture. Whereas, alveolar and cortical bone showed the lowest viability readings under both time points.

For glucose consumption, we were looking at glucose levels collectively in the conditioned media, isolated form organ cultures at 4 time points (overnight controls (D0), day1, day 2, and day4), which offered an indication of the overall viability of the whole tooth *in situ* organ culture, at the different time points of the culture, rather than the viability of the individual tissues. There was no significant difference in glucose consumption conditioned medium obtained at the 4 different time points (overnight control, 1 day, 2 days, and 4 days) tooth *in situ* samples were incubated overnight (controls) and conditioned medium obtained from tooth *in situ* organ cultures after 4 days (Figure 5B). However, there was a dip in the glucose consumption at day 2 compared to controls, day 1 and day 4. Whereas, the highest percentage of glucose consumption was observed at day 1.

### Comparing Histologic Appearance of Tooth *in situ* Organ Cultures to Freshly Extracted Control Tissues

**Figure 6:**
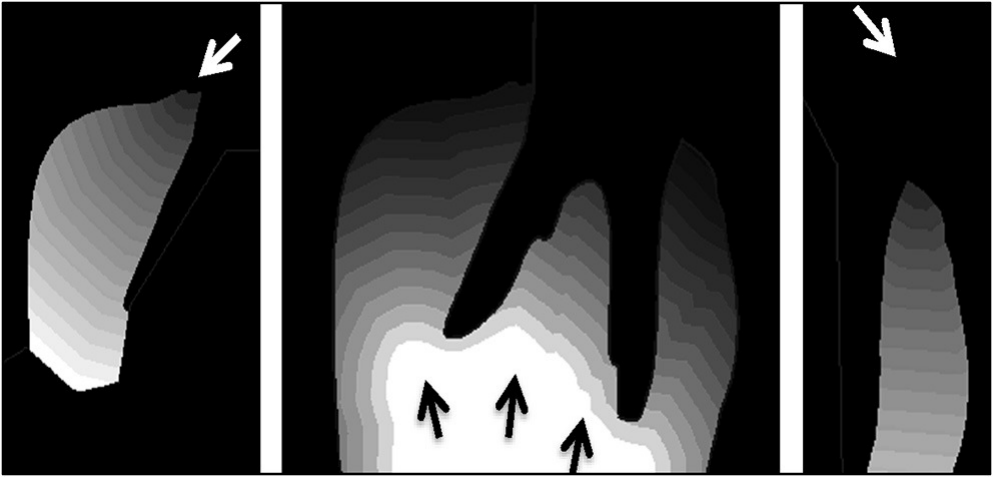
Contour tissue depth map demonstrating the distance media will need to diffuse into tissue from the three media entry points shown separately **(left, middle, and right)**. Each contour line depicts a depth of 1 mm from the media entry (arrow).

We checked for any evidence of tissue necrosis associated with the methods used in organ culture of the tooth *in situ* samples after 4 days using histological assessment. Light microscopy images of sections of tooth *in situ* samples stained with haematoxylin and eosin showed no visible signs of tissue necrosis after organ culture for 4 days. The histological appearance of both the alveolar and cortical bone of the lingual and buccal plates of the mandible was apparently unchanged after culture compared to freshly prepared controls (Figures 6A–F). Periodontal ligament thickness and structure in the tooth *in situ* samples after 4 days of organ culture were also similar to those of freshly prepared controls (Figures 6E,F). Bone and periodontal structure within the molar root bifurcation area were apparently healthy for tooth *in situ* samples after 4 days of organ culture and were directly comparable in appearance of the freshly prepared controls (Figures 6G,H). Gingival tissue was seen to be intact and similar in samples obtained from organ culture and freshly prepared controls but the outermost keratinised epithelial layer had been apparently lost in the tooth *in situ* samples that had undergone organ culture, which may have been due to sample manipulation during histological preparation (Figures 6I,J).

### Histomorphometric Analysis of Tooth *in situ* Samples in Comparison to Freshly Extracted Control

To provide a more detailed and quantitative comparison between tissues in control and tooth *in situ* samples after 4 days of organ culture, we carried out histomorphometic analyses of the histological sections as described previously in section Modeling Diffusion Based on Tissue Porosity. There were no significant differences between the average number of cells in a 1 mm^2^ field of PDL and alveolar bone in the *in situ* samples cultured for 4 days compared to fresh controls. The average number of blood vessels and the average thickness of the PDL at mid-root also showed no significant difference between 4 days organ cultures and fresh controls. However, the number of cells and the thickness of the PDL showed an increase in the tooth *in situ* organ culture compared to the fresh controls. Nevertheless, the number of blood vessels in PDL tissues were slightly less in the organ culture compared to fresh controls. Whereas, the number of cells and blood vessels in bone was very similar both in the organ culture and the fresh controls. Histomorphometric analysis data are summarized in Table 1.

**Table 1:** Histomorphometric analysis for histological sections of fresh controls and organ culture tooth *in situ* samples cultured for 4 days.

**Criteria of assessment**	**Fresh Control**	**Organ culture tooth *in situ* (4 days)vv**	**Statistical significance**
Average Cell count in 1 mm^2^ of PDL	148.3 ± SD (26.23)	158.7 ± SD (26.08)	NS
Average cell count in a 1 mm^2^ of bone	24.6 ± SD (3.01)	24.17 ± SD (4.6)	NS
Average blood vessel counts in 1 mm^2^ of PDL	2.5 ± SD (1.05)	1.8 ± SD (0.75)	NS
Average blood vessel counts in 1 mm^2^ of Bone	1.6 ± SD (0.5)	1.5 ± SD (0.54)	NS
Average PDL thickness at mid root in μm	1079.6 ± SD(175.6)	1124.6 ± SD (284.4)	NS

### Modeling of Perfusion of Tooth *in situ* Tissues in Organ Culture

**Figure 7:**
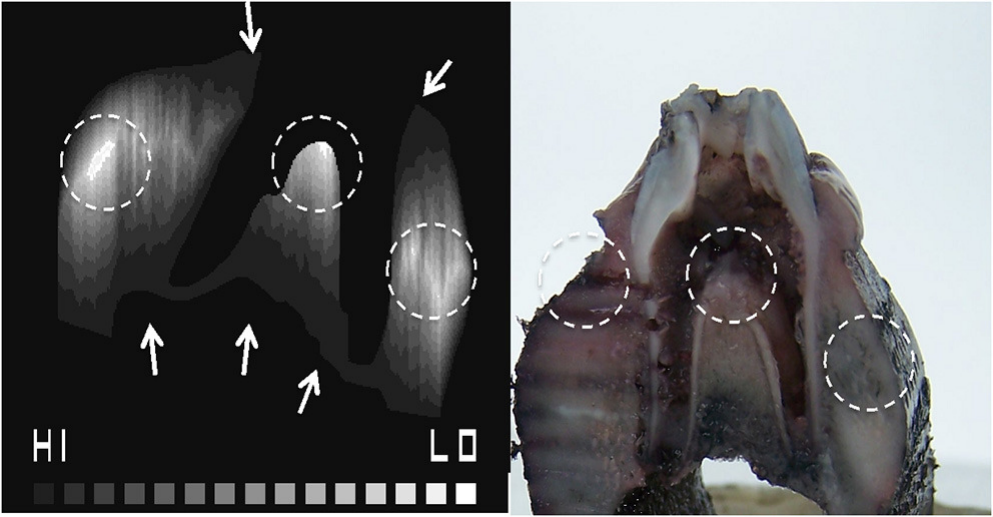
Combined porosity contour map identifying hard to reach areas for diffusion where the HI to LO scale indicates predicted high to low diffusion, respectively **(A)**. The Indian ink diffusion test demonstrating areas of low diffusion **(B)**, which matches the porosity contour map (dotted circles).

**Figure 8:**
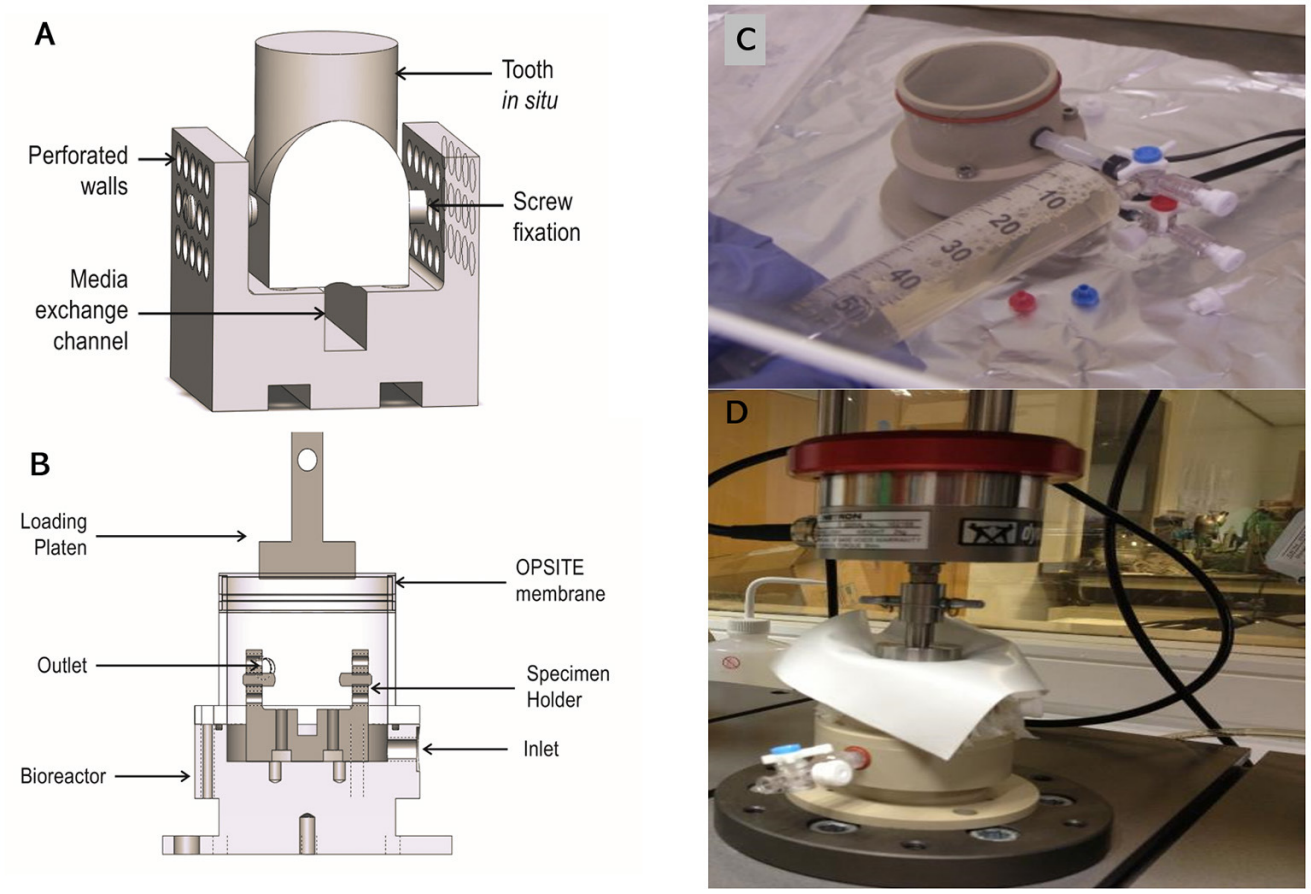
Schematic of the bioreactor design capable of accommodating a single porcine first molar *in situ*. The tooth in situ is seated on the specimen holder **(A)**. Silicone tubes connect the inlet and outlet allowing for gases and medium to flow **(B)**. A photograph of the bioreactor sealed with OPSITE®, membrane to maintain culture sterility, and showing inlet and outlet required for changing media under aseptic conditions **(C)**. A photograph of bioreactor under loading regime replicating daily chewing activity **(D)**. The preloading regime was −5 to −45 N sinusoidal wave at 0.25 Hz for 10 cycles and the loading regime was −5 to −100 N at 1 Hz for 1,400 cycles.

The porosity map from the micro-CT image (Figure 2) and the contour tissue depth map (Figure 7) were combined to produce a predicted tissue diffusion map (Figure 8). Regions with the thickest cortical bone and the bifurcation area indicate the slowest diffusion rates or the furthest areas for medium to reach (Figure 8). The tissue diffusion map predicted that the tooth *in situ* samples would be perfused with medium, though at different rates within the same tissue (Figure 8). Regions with the thickest cortical bone, together with the bifurcation area, were predicted to have the slowest diffusion rates and/or were the furthest areas for medium to reach. The Indian ink diffusion test indicated complete diffusion through the gingiva after 18–21 h and confirmed regions of slow diffusion that were similar to the tissue diffusion map (Figure 8).

### Testing the Bioreactor Unit

The bacterial swabs that were taken at different locations and components of the system (silicon loading cap, inlet, outlet, specimen holder, and bioreactor chamber) for aerobic and anaerobic bacterial culture showed no growth, confirming sterility.

**Figure 9:**
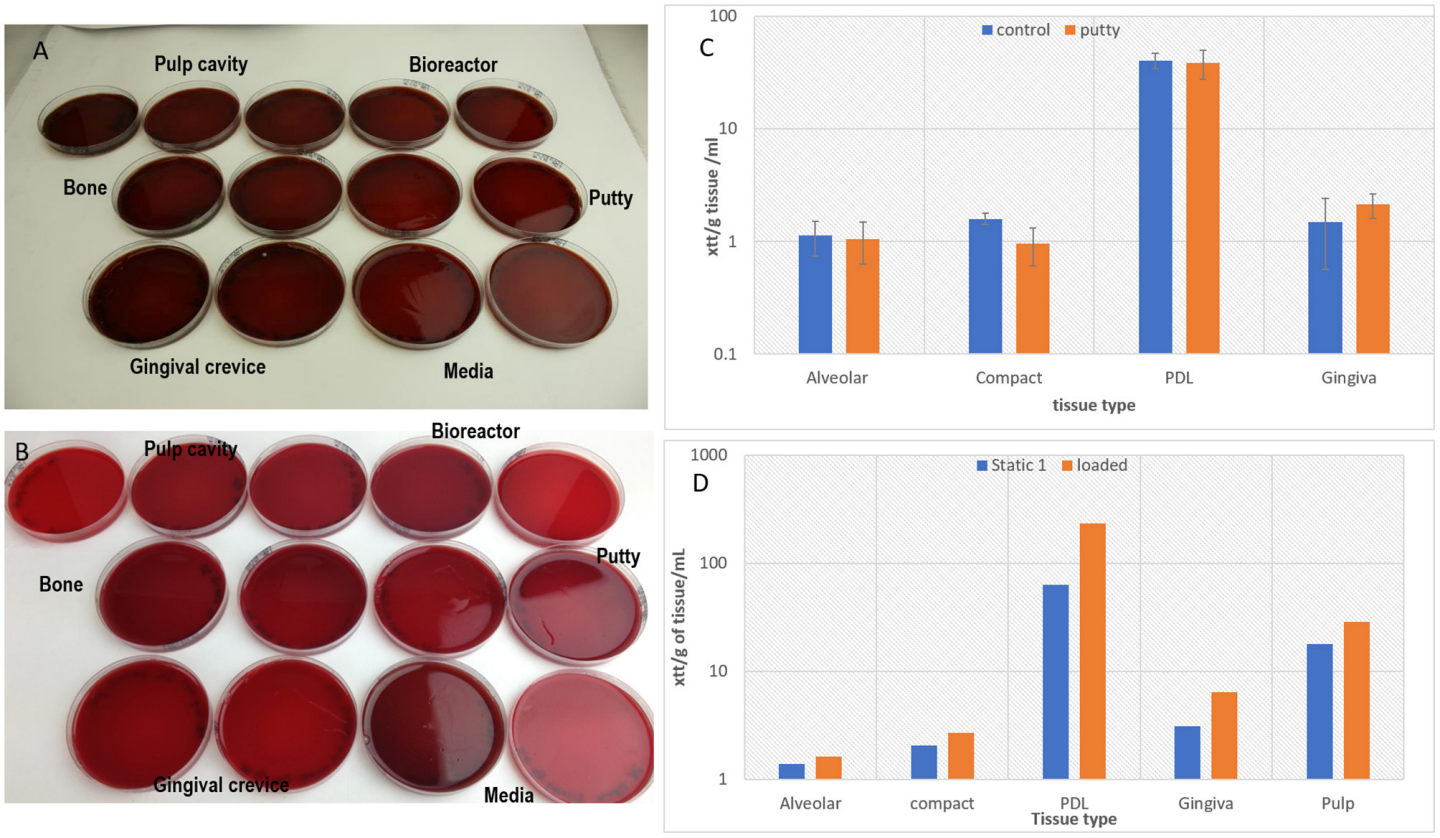
Showing clear Aerobic **(A)** and anaerobic **(B)** bacterial cultures of swabs (samples) taken from the different parts of bioreactor and from media and from tissues confirming sterility after 4 days of culture during which the loading regime has been applied. The tissue viability was confirmed after 4 days of static culture in the bioreactor presence of the putty cap and absence of putty for the tooth *in situ* model (*n* = 3) **(C)**, and a graph confirming cell viability after applying the loading regime in the bioreactor and maintaining the culture for 4 days (*n* = 1) **(D)**.

The loading cap integrity was tested on a tooth structure using the loading regime in a non-sterile test showed the silicon loading cap was structurally undamaged by the loading (Figure 9), tested samples using the putty cap in static culture without loading was confirmed to maintain all tissues viability compared to cultures without the putty cap (Figure 9C).

One *in situ* sample was successfully loaded and maintained sterility against aerobic (Figure 9A) and anaerobic (Figure 9B) bacteria and tissue viability in comparison to unloaded samples (Figure 9D). However, there were incidences of infections with aerobic bacteria in some of the loading experiment particularly in swabs taken from the putty cap and from the gingival crevice (results not shown).

## Discussion

A regenerative approach to the treatment of PD has been shown to be potentially superior to current non-regenerative clinical treatments (Dentino et al., 2013). However, to carry out the pre-clinical investigations that are necessary to develop innovative technologies for the regeneration of the periodontium, as well as testing osseointegration of implants, a suitable model needs to be available. An *ex-vivo*, whole tooth, *in situ*, 3D organ culture model that maintains the complexity and inter-relationships of the very different tissues of the tooth and its periodontium and that could be subjected to application physiological-like loading would be ideal. Such a model would ideally use teeth that are as close as possible in terms of their size and architecture to human teeth. However, development of such a model faces major challenges in the maintenance of tissue viability and especially in achieving sterility, given that the mouth is a non-sterile environment, and the microbial load is very high.

Our main objectives in developing the proposed porcine tooth *in situ* model were to maintain tissue viability for all the different tissues in the periodontium and surrounding bone and to keep the organ culture free from microbial contamination throughout a short term culture period of 4 days. A methodology was successfully developed to deliver to this, comprising of a series of washing steps, pre-culture in media and antibiotic selection, based on knowledge of the periodontal microbiome, of an optimized antibiotic cocktail that was shown to maintain sterility of organ cultures in 90% of samples over a 4 day culture period.

In order to determine tissue viability within the organ culture samples, we used both XTT assays to determine metabolic activity within the individual tissue components of the periodontium together with an assay to determine glucose consumption in the organ culture medium. This latter assay was developed not only as validation for the XTT results, which would be affected by any microbial contamination but also as a prelude to future online monitoring of viability when the organ culture model is transferred to a bioreactor that will apply physiological-like loading (Elson et reference, see before). The histological appearance of the tooth *in situ* samples after 4 days of organ culture further confirmed the viability of the model, with no signs of necrosis in any of the tissues (osteoclastic activity was detected in both freshly prepared controls and in the organ culture samples).

Sloan et al. (1998) pioneered organ culture for the dental tissues in successfully developing a rat incisor tooth slice model that was cultured for 14 days at the air-liquid interface, showing that the dentine-pulp complex remained morphologically intact throughout the culture period. Furthermore, dentine formation was observed within the model, indicating viability and normal histogenesis (Sloan et al., 2013) However, this early model did not include mandibular bone or periodontal ligament and was therefore not appropriate for use in periodontal applications. Smith et al. (2010) developed an *ex vivo* rat mandible slice model in order to investigate bone remodeling and repair and included the periodontal ligament. Maintenance of the different cell types and tissue structure was observed over a 21 day culture period but there was a significant drop in cell numbers after 21 days (Smith et al., 2010). Both models, in spite of being successful in the study of bone repair, were based on thick slices rather than intact teeth *in situ* within the mandible and were dis-similar to human teeth and periodontium in terms of periodontal physiology as rodents have a characteristic gnawing habit. Another disadvantage of these rodent models for our own long term aim was the size of the tooth/or mandibular slice which was only 2 mm compared with our own samples which were circa 30 mm in thickness, more closely resembling the human situation, in spite of the short term culture period achieved.

The very strength of our tooth *in situ* porcine model in terms of its size and more faithful representation of dental physiology to tissue orientation and spatial organization, presented a huge challenge in terms of maintenance of tissue viability in culture. We chose to use porcine teeth because these have a general size, morphology, mastication force and PDL physiology i.e., similar to that of humans and pigs also develop spontaneous PD (Bousdras et al., 2006; Sonoyama et al., 2006; Liu et al., 2008). In addition, these teeth are relatively easy to source immediately after slaughter of the animals for food, consistent with the principles in reducing, refining, and replacing animals bred specifically for research purposes.

Furthermore, porcine bone tissues are similar to human tissues, in their osteogenic gene profile which made them a suitable candidate for clinical use as xenograft for alveolar bone augmentation and regeneration in socket healing and grafting pre-implant insertion (Crespi et al., 2011a,b).

In order to further our understanding of the potential for using the porcine tooth *in situ* in extended culture periods we developed methodologies for online modeling and for 3D imaging and diffusion modeling which have not previously been used as an adjunct for organ culture of intact teeth and the periodontium. As *ex vivo* tissue maintained in organ culture depends on diffusion of nutrient and gaseous exchange, the greater the tissue thickness, the higher the risk of tissue necrosis, resulting in cell death. Diffusion rates of tissue culture medium were expected to vary with bone porosity; therefore a combined contour porosity map was created that took into account diffusion rates from the gingival tissues and the mandibular canal. The porosity map was derived from a CT image slice (Figure 2) where the greyscale value of bone in CT images is directly proportional to mineral density and is a good indicator of bone porosity (Cooper et al., 2007).

The predictive value of the 2D tissue diffusion map was validated by testing the diffusion of Indian ink with 3D porcine samples in our culture system. The contour porosity mapping used to predict the tissue diffusion map, predicted the areas of slow diffusion revealed in the subsequent ink test (Figure 8). This provided some confidence that contour porosity mapping can predict the diffusion pathways within the gingival tissue and bone. This method takes account of the two physical parameters that influence diffusion: distance to the external medium (tissue thickness) and porosity (derived from bone density). However, the diffusion map does not consider concentration gradients or chemical interactions between tissues and fluids.

The complete perfusion of the gingival tissues seen in the ink test and modeled in our tissue diffusion map correctly predicted that there would be no problems with cell viability due to lack of nutrient from the medium in our tooth *in situ* model. The viability data obtained from these tissues compared with non-cultured controls confirmed that this was indeed the case. Our models also predicted that the dense cortical bone tissue, where cell density is low, would experience slow rates of diffusion that might prove problematic were the culture periods to be extended. There was no obvious loss of viability in these areas over the 4 days used in our studies.

The availability of a relevant intact tooth *in situ* organ culture model is an essential pre-requisite to moving into a bioreactor environment to complete any physiological simulation and so test any regenerative interventions in the model. Physiological loading of the tooth is known to be of a paramount importance for the health, homeostasis and remodeling of both the PDL and the alveolar bone (Jiang et al., 2016; Chukkapalli and Lele, 2018). The available tooth and mandibular slice models had lacked this element of physiological loading (Sloan et al., 1998; Pavlin and Gluhak-Heinrich, 2001; Mavropoulos et al., 2005; Milne et al., 2009; Choi et al., 2011). However, the mandibular slice system developed by Smith et al. (2010), was recently modified to include application of orthodontic tensile loading using an arch wire spring (Wan Hassan et al., 2012). The group also investigated the use of low intensity pulsed ultrasound to stimulate bone and PDL remodeling under orthodontic forces (El-Bialy et al., 2011). Other studies attempted to simulate the periodontal complex using PEAK (polyaryletherketone) to represent the bone and the periodontal ligament to investigate the effect of mechanical loading on PDL cells *in vitro* (Berendsen et al., 2009), and there have been reports investigating the effect of stretching on PDL cells in a monolayer system (Oortgiesen et al., 2012). Such models may be suitable for tissue engineering constructs or investigating the effect of mechanical loading at the cellular level, but they are unsuitable as simulation models. Our group has recently developed a bioreactor capable of loading our porcine tooth *in situ* to simulate normal orgnathic forces physiological forces, including programmable chewing cycles. Our next step will be to transfer the organ culture to this bioreactor.

The reoccurrence of infection in some cases after applying the loading regime, might have been induced by the mechanical loading as found clinically in cases of marginal leakage of dental restorations (Khvostenko et al., 2015). However, we were able to successfully load one sample in the bioreactor whilst maintain sterility and tissue viability.

Long term culture might be a limitation in our porcine tooth *in situ* model due to the anatomical size and complexity of the tissues, this remains to be tested. However, the data presented here is promising for the further development of the model to investigate tissue engineering and regenerative strategies for periodontal repair and even Osseo integration of dental implants. Simple modifications to the loading regime and slight structural modification of this bioreactor will expand the use of this bioreactor to other applications such as *in vitro* orthodontic models, bone regeneration models, and biomaterials testing.

## Conclusion

A novel porcine tooth *in situ* organ culture model has been developed. Sterility, tissue viability and structural morphology of the tissues were maintained for 4 days in organ culture. In parallel, *in silico* modeling of the diffusion pathways in the tissues proved capable of predicting areas of slow and rapid perfusion. A bioreactor has been designed to accommodate the organ culture and allow future loading. Future perspectives for this model include testing regenerative therapies of bone and PDL including stem cell-based therapies, in addition to investigating stem cell recruitment and cellular responses to inflammation and biomaterials.

## Data Availability Statement

The raw data supporting the conclusions of this article will be made available by the authors, without undue reservation.

## Ethics Statement

Ethical review and approval was not required for the animal study because we have used animal porcine cadaveric heads from the slaughterhouse and this didn't require any ethical approval.

## Author Contributions

RE-G, SJ, SL, and KE contributed to study design, lab work, and manuscript writing and revision. RE-G, SJ, and SL are equal contributors to this manuscript as first authors. JT, RH, EI, and JK contributed to study design and manuscript writing and revision. All authors agree to be accountable for the content of the work.

## Conflict of Interest

The authors declare that the research was conducted in the absence of any commercial or financial relationships that could be construed as a potential conflict of interest.
